# The Immune Checkpoint Landscape in Tumor Cells of Pancreatic Ductal Adenocarcinoma

**DOI:** 10.3390/ijms24032160

**Published:** 2023-01-21

**Authors:** Florian N. Loch, Carsten Kamphues, Katharina Beyer, Christian Schineis, Wael Rayya, Johannes C. Lauscher, David Horst, Mihnea P. Dragomir, Simon Schallenberg

**Affiliations:** 1 Department of Surgery, Charité—Universitätsmedizin Berlin, Corporate Member of Freie Universität Berlin and Humboldt-Universität zu Berlin, 12203 Berlin, Germany; 2 Department of Surgery, Park-Klinik Weißensee, 13086 Berlin, Germany; 3 Institute of Pathology, Charité—Universitätsmedizin Berlin, Corporate Member of Freie Universität Berlin and Humboldt-Universität zu Berlin, 10117 Berlin, Germany; 4 Berlin Institute of Health at Charité, Charitéplatz 1, 10117 Berlin, Germany; 5 German Cancer Research Center (DKFZ), German Cancer Consortium (DKTK), Partner Site Berlin, 69210 Heidelberg, Germany

**Keywords:** pancreatic ductal adenocarcinoma, immune checkpoints, survival, immune checkpoint inhibitors, immune checkpoint treatment

## Abstract

Immune checkpoint therapy (ICT) has shown promising potential in the treatment of multiple solid tumors. However, the role of ICT in pancreatic ductal adenocarcinoma (PDAC) remains limited. Patterns of immune checkpoints (ICs) in PDAC represent the basis for establishing a potent ICT. The aim of this study is to create a profile of IC expression and its prognostic relevance in cancer cells of PDAC. Therefore, tumor cells from peripheral and central tissue microarray (TMA) spots from histologically confirmed PDAC of 68 patients after tumor resection were investigated in terms of expressions of TIM3, IDO, B7H4, LAG3, VISTA, and PD-L1 using immunohistochemistry. The presence of the respective ICs was compared to overall survival (OS). The presence of VISTA and PD-L1 significantly correlates with shorter OS (median OS: 22 months vs. 7 months and 22 months vs. 11 months, respectively, *p* < 0.05). For the presence of TIM3, IDO, B7H4, and LAG3, no difference in OS was observed (*p* > 0.05). The analysis of OS of combined subgroups for VISTA and PD-L1 (VISTA and PD-L1 neg., VISTA pos. and PD-L1 neg., VISTA neg. and PD-L1 pos., and VISTA and PD-L1 pos.) yielded overall statistical significance difference (*p* = 0.02). These results suggest that the presence of VISTA and PD-L1 is of prognostic relevance and potentially qualifies them as targets for ICT.

## 1. Introduction

Immune checkpoints (ICs) are molecules involved in the stimulation and inhibition of the interaction of antigen-presenting cells (APC) and cells of the immune system, and thus they play a crucial role in the regulation of immune responses. The interaction between cancer cells and immune cells through ICs in the tumor microenvironment (TME) inhibits immunosurveillance and mediates the immune escape of the tumor. Thereby, ICs promote tumor development, growth, invasion and metastasis [1]. More specifically, the interaction of ICs in the TME caused by produced soluble ligands or as membrane-bound ligands of an APC—such as a cancer cells and receptors of immune cells, mainly CD4+ helper T-cells, and CD8+ effector T-cells—ultimately suppresses immune responses [2]. By inhibiting these interactions, immune checkpoint therapy (ICT) with immune checkpoint inhibitors (ICI) has shown outstanding potential in the treatment of multiple solid tumors [3]. Programmed cell death protein (PD-1) and programmed death-ligand (PD-L1) inhibitors are most established and have been approved for the therapy of advanced melanoma, metastatic non-small cell lung cancer and multiple other types of cancer [3]. 

Approximately 466,000 patients die yearly from pancreatic ductal adenocarcinoma (PDAC) worldwide [4], and the overall 5-year survival after diagnosis remains 9%, despite tremendous efforts to improve early detection and clinical management [5,6]. However, despite its aggressiveness, the role of ICIs in PDAC has not been thoroughly elicited, and thus currently remains limited [7]. An understanding of the IC patterns of a specific tumor entity and their clinical relevance represents the basis for the development, identification and establishment of a respective potent ICT. The ICs—PD-L1, Indoleamine 2, 3-Dioxygenase (IDO) and V-set domain-containing T-cell activation inhibitor 1 (B7H4)—are known to potentially be expressed in cancer cells with the capability to inhibit immune responses by interaction with immune cells [2,8,9]. Studies investigating the role of the ICs T-cell immunoglobulin and mucin domain 3 (TIM3), lymphocyte-activation gene 3 (LAG3) and V-domain immunoglobulin suppressor of T-cell activation (VISTA) mainly focus on their expression on immune cells, especially T-cells, in the TME [2,10,11,12,13]. However, there are indications that TIM3, LAG3 and VISTA expressions in cancer cells lead to an immunosuppressive TME, is of prognostic relevance, and thus a potential target for future ICIs [14,15,16].

Thus, the aim of this study is to understand the landscape of IC in resected PDAC by investigating the presence of TIM3, IDO, B7H4, LAG3, VISTA, and PD-L1 in cancer cells and to determine their prognostic relevance in terms of overall survival (OS). 

## 2. Results

### 2.1. Demographics, Clinicopathological Characteristics and Survival of Patient Cohort

The demographic and clinicopathological characteristics of the patient cohort are shown in Table 1. Median survival of the entire patient cohort was 17 months (CI: 9.9–24.1, range 1–118). The one-year, three-year, and five-year survival rates of the entire patient cohort were 60.3%, 26.5% and 19.1%, respectively. 

Table 2 shows hazard ratios for OS in univariate and multivariate analysis for the respective demographic and pathological characteristics. For the nodal involvement characteristics (pN+ vs. pN0), resection margin (R1 vs. R0) and angioinvasion (pV1 vs. pV0), hazard ratios showed significance in univariate and multivariate analyses (*p* < 0.05). The hazard ratio of lymphatic vessel invasion (pL1 vs. pL0) showed significance only in the univariate analysis (*p* < 0.05).

### 2.2. Presence of TIM3, IDO, B7H4, LAG3, VISTA and PD-L1 in Cancer Cells of PDAC

**Figure 1:**
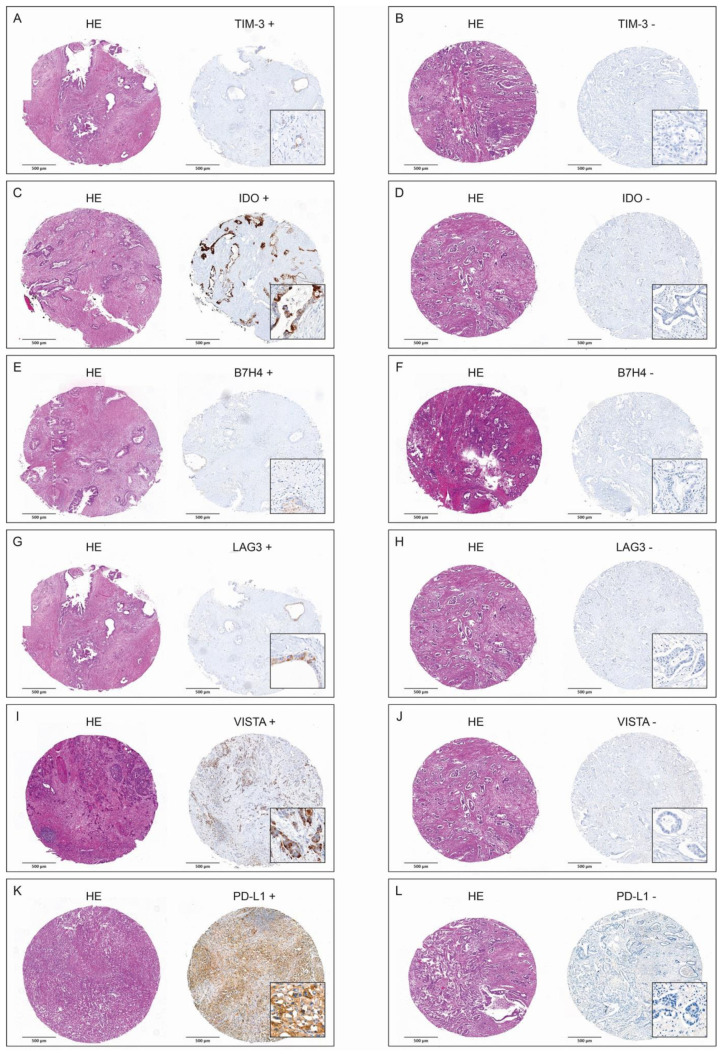
H&E-stained tumor spots and corresponding positive and negative IHC stainings of TIM3 (**A**,**B**), IDO (**C**,**D**), B7H4 (**E**,**F**), LAG3 (**G**,**H**), VISTA (**I**,**J**), and PD-L1 (**K**,**L**).

At least one of the ICs—TIM3, IDO, B7H4, LAG3, VISTA or PD-L1—was present in cancers cells in 66.2% (n = 45) of resected tumors. IDO was most frequently present (n = 28, 41.2%), and TIM 3 was least frequently present (n = 11, 16.2%). Table 3 shows the presence of the respective ICs in the entire study group, as well as individually in the subgroups of survivors and non-survivors. VISTA and PD-L1 were present in cancer cells of 30.9% (n = 17), and 38.2% (n = 21) of the resected tumors in the subgroup of non-survivors and only in 7.7% (n = 1) and 15.4% (n = 2) of tumors in the subgroup of survivors, respectively. Representative examples of presence and absence of TIM3, IDO, B7H4, LAG3, VISTA and PD-L1 in immunohistochemical (IHC) staining are presented in Figure 1A–L.

### 2.3. Correlation of Overall Survival and TIM3, IDO, B7H4, LAG3, VISTA, and PD-L1 in Cancer Cells of PDAC

#### 2.3.1. General Analysis

**Figure 2:**
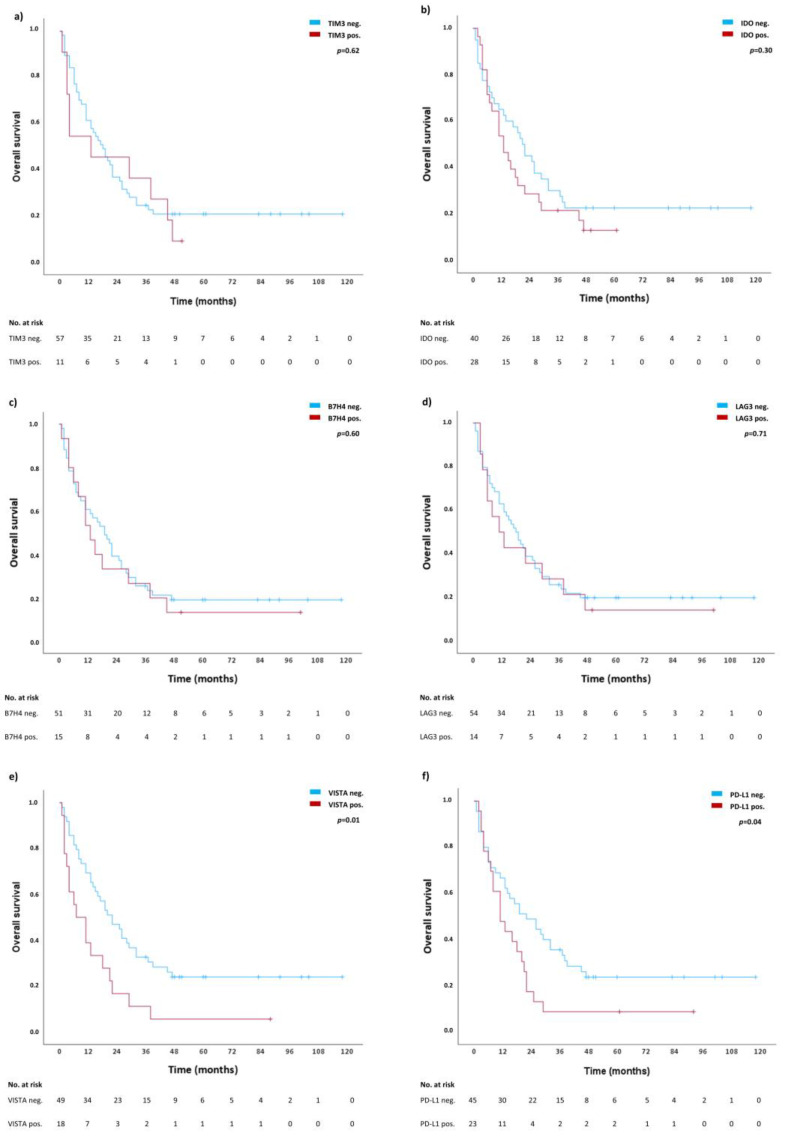
Individual survival analyses for TIM3 (**a**), IDO (**b**), B7H4 (**c**), LAG3 (**d**), VISTA (**e**), and PD-L1 (**f**). Blue lines represent survival curves in absence of the respective ICs, whereas red lines represent survival curves in presence of the respective IC. Tick marks indicate censoring.

Figure 2 shows the result of individual survival analyses for the expression of TIM3 (Figure 2a), IDO (Figure 2b), B7H4 (Figure 2c), LAG3 (Figure 2d), VISTA (Figure 2e), and PD-L1 (Figure 2f) on tumor cells. The presence of VISTA and PD-L1 significantly correlates with shorter OS (median OS in VISTA neg. subgroup 22 months (CI: 12.9–31.1 months) vs. 7 months (CI: 3.5–13.9) in VISTA pos. subgroup, *p* = 0.01, and 22 months (CI: 10.2–33.8) in PD-L1 neg. subgroup vs. 11 months (CI: 5.1–16.9) in PD-L1 pos. subgroup, *p* = 0.04). For the presence of TIM3, IDO, B7H4 and LAG3 in tumor cells, no difference in OS was observed (*p* > 0.05).

#### 2.3.2. Combined Analysis of Significantly Correlated Immune Checkpoints VISTA and PD-L1 

**Figure 3:**
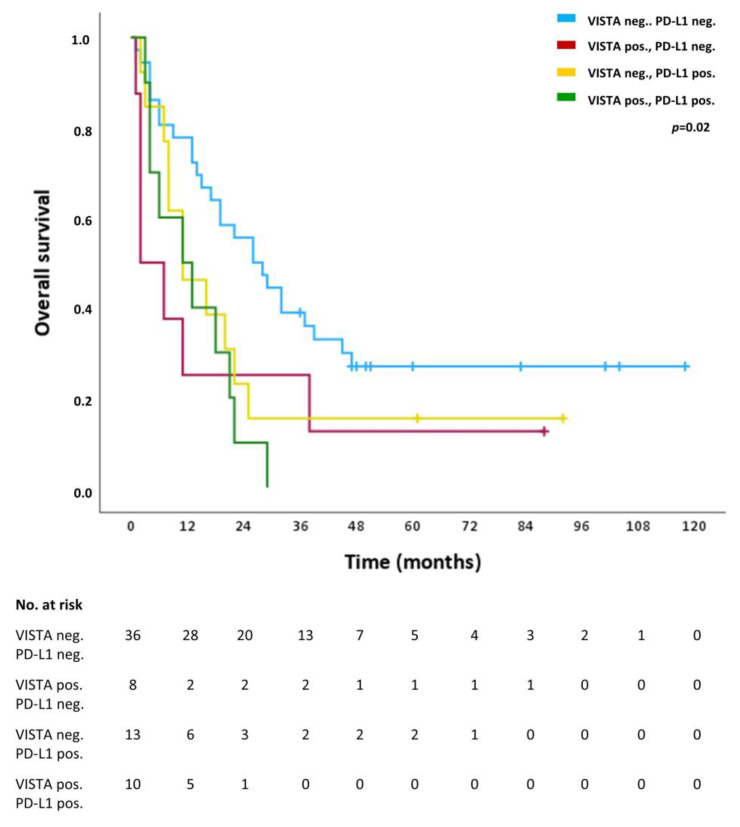
Overall survival (OS) of the subgroups VISTA neg. and PD-1 neg. (blue line), VISTA pos. and PD-L1-neg. (red line), VISTA neg. and PD-L1 pos. (turquoise line), and VISTA pos. and PD-L1 pos. (purple line). Tick marks indicate censoring.

For VISTA and PD-L1 an in-depth subgroup analysis was performed correlating the absence of both VISTA and PD-L1 (VISTA neg., PD-L1 neg.), the presence of either VISTA (VISTA pos., PD-L1 neg.) or PD-L1 (VISTA neg., PD-L1 neg.) or both VISTA and PD-L1 (VISTA pos., PD-L1 pos.) with OS. Table 4 shows the presence of the respective combination of ICs in patients as well as one-year, three-year and five-year survival rates for each subgroup. The OS rates of the subgroup VISTA neg., PD-L1 neg. are the highest, whereas the OS rates of the subgroup VISTA pos., PD-L1 pos. are the lowest, intermediated by the subgroups with one of the ICs being present (VISTA pos., PD-L1 neg. and VISTA neg., PD-L1 pos.). Figure 3 shows the survival curves of OS for the combined analysis for all four subgroups with overall statistical significance difference (*p* = 0.02). Consistent with the survival rates, the survival curves of the subgroup VISTA neg., PD-L1 neg. and the subgroup VISTA pos., PD-L1 pos. diverge the most and are intermediated by subgroups with positivity for one of the ICs (VISTA pos., PD-L1 neg. and VISTA neg., PD-L1 pos.).

### 2.4. Heterogeneity of VISTA and PD-L1 in Cancer Cells of PDAC

**Figure 4:**
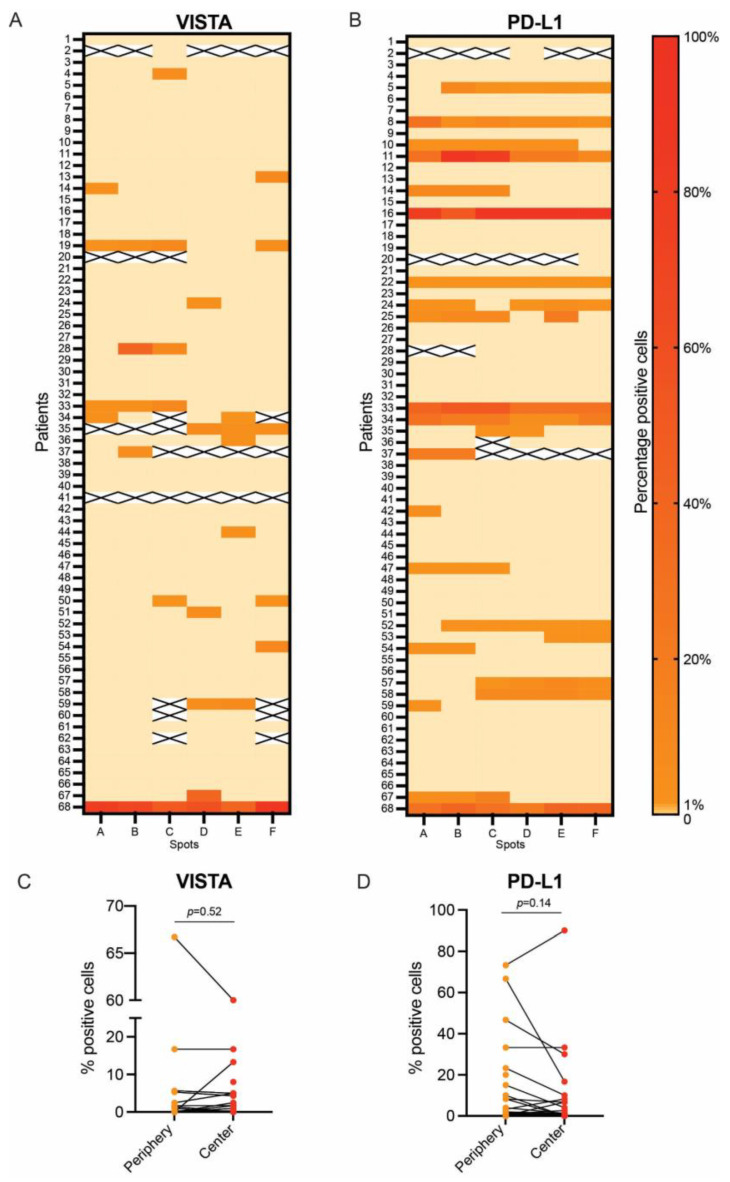
Intratumoral heterogeneity of VISTA and PD-L1 expression: percentage of VISTA expression in tumor cells across the entire PDAC cohort (**A**) and percentage of PD-L1 expression in tumor cells across the entire PDAC cohort (**B**). Paired comparison of VISTA expression in spots of tumor periphery vs. spots of tumor center (**C**) and paired comparison of PD-L1 expression in spots from tumor periphery vs. spots from tumor center (**D**).

For the two ICs that significantly correlated with shorter OS, we analyzed their distribution across the different spots of the TMA and compared their expressions between tumor periphery vs. tumor center. For VISTA, we observed an intratumoral heterogeneity: Of the 18 patients expressing VISTA, two (11.1%) were positive in all spots (100%), one patient (5.6%) in 66.7% of the spots, four patients (22.2%) in 50% of the spots, two patients (11.1%) in 33.3% of the spots, and the majority (n = 9, 50%) in one of six spots (16.67%, Figure 4A). PD-L1 had a higher degree of homogeneity regarding its expression: Eight patients (34.8%) showed positivity in 100% of the analyzed spots, four patients (17.4%) in 83.3% of the spots, three patients (13.0%) in 66.7% of the spots, three (13.0%) in 50.0% of the spots, another three (13.0%) in 33.3% of the spots, and only two patients (8.7%) in 16.7% of the spots (Figure 4B).

The expressions of VISTA and PD-L1 were also compared to the average expression of spots localized at the periphery of the tumor vs. average expression of spots localized in the center of the tumor. This paired analysis showed no difference in the expressions of VISTA (*p* = 0.52, Figure 4C) or PD-L1 (*p* = 0.14, Figure 4D) in the periphery vs. the center of PDAC.

Additionally, survival analyses were performed for subgroups of VISTA and PD-L1 expression (1) only in the periphery and (2) only the center of tumors as well as (3) expression in both, the periphery and the center of the tumor. For PD-L1 there was no significant impact on OS for these individual subgroups (*p* > 0.05). VISTA expression in the center only and in both the center and the periphery of tumors showed a significant correlation with shorter OS (*p* < 0.05), whereas expression only in the periphery of tumors does not correlate with shorter OS (Table 5). Two of the eighteen patients (11.1%) expressing VISTA were excluded from this analysis due to absence of peripheral tumor spots in the TMA.

## 3. Discussion

From a mutational point of view, PDAC is a very homogenous malignant disease. This cancer type is defined by frequent *KRAS*, *TP53*, and *SMAD4* mutations [17], making DNA sequencing data far less valuable for patient prognosis and therapy selection. Hence, our aim was to discover new markers that can stratify patients with PDAC and reveal new potential therapeutic targets.

ICs are established prognostic and therapeutic targets in multiple malignancies [18] and thousands of clinical trials are ongoing to broaden our arsenal of inhibitors for multiple cancer types, including PDAC [19]. The only ICT approved for PDAC is the agnostic approval of anti-PD1 for tumors that are MSI-H/dMMR. PDACs with MSI-H/dMMR represent only 0.8–2% of all PDACs, and even for these tumors, the response rate is limited [7]. PDAC is perceived as a “cold” malignancy from an immunological point of view with a low mutational burden making ICT very challenging. Therefore, we believe that a deeper biological understanding of ICs in these tumors is crucial and future therapeutic regimes are likely to contain combinations of ICTs that target multiple immune checkpoint proteins.

We used a well-annotated cohort of PDAC patients, for whom we had long-term survival data, and analyzed the expression levels of TIM3, IDO, B7H4, LAG3, VISTA and PD-L1 in cancer cells by IHC to obtain an overview of IC expression in this neoplasia. We observed that PD-L1- and VISTA-positive tumors are associated with shorter OS. Regarding PD-L1 expression, our results are in line with previous studies. In a cohort of 373 patients with PDAC, Linag et al. showed that PD-L1 positivity in tumor cells is associated with shorter OS and progression-free survival. Additionally, they observed a borderline association between PD-L1 positivity and lymph node metastases [20]. In a more recent paper, this observation was also confirmed: PD-L1 positivity in tumor cells in PDAC, but not in immune cells, correlates with a shorter OS [21].

The role of VISTA as a prognostic marker in PDAC is much more controversial. In a large study, Hou et al. analyzed the expression of VISTA in two cohorts of PDAC. They observed that high VISTA expression is borderline associated with longer OS compared with the low expression of VISTA in both cohorts. It is important to note that Hou et al. methodologically divided VISTA into high-expression vs. low-expression groups and not positive vs. negative, which was the case in this study [22]. The results of the subgroup analysis of our study show a loss of significance regarding the negative effect on OS for expression of VISTA in ≥10% of tumor cells (Supplementary Table S2). This is because, in our study, the negative effect on median survival does not increase with a higher percentage of positive tumor cells, whereas the number of cases in which VISTA is present to such a high extent decreases. Additionally, our results show that VISTA expression is heterogeneous within tumors and only has an impact on OS in cases where it is present in the center of the tumor. For VISTA, in contrast to PD-L1, there is currently no consensus regarding what staining interpretation is the most clinically relevant, and this point needs to be addressed in future studies. However, our results underline the importance of sampling multiple regions in order to discover VISTA positivity and indicate that the center of tumors may present a TME, in which VISTA expression on tumor cells has a prognostic impact, in contrast to the periphery of tumors. Hou et al. also analyzed the expression of VISTA in immune cells and endothelial cells but did not observe any differences in OS [22].

Another study, complementary to ours, analyzed the expressions of IDO, VISTA, LAG3, and TIM3 in tumor-infiltrating lymphocytes in PDAC patients from the PANCALYZE study cohort. Here, Popp et al. observed that a high expression of IDO, but not of VISTA, LAG3, and TIM3, is associated with a prolonged OS [10]. In our study, however, a significant negative effect on survival in presence of IDO in ≥10% and <50% of tumor cells was found (Supplementary Table S2, median survival 19 months (CI: 12.4–25.6) vs. 4 months (CI: 0.0–13.2), *p* = 0.016). This agrees with existing studies that identify a strong expression of IDO in cancer cells as a negative prognostic factor [23]. These complementary data point out the versatile role that IC molecules can play and the differences in their significance between immune cells and tumor cells.

Finally, by performing a combined analysis, we observed that negativity in both VISTA and PD-L1 characterizes a subgroup of patients with significantly longer OS, while positivity in both is associated with the worst OS.

Our study has limitations that need to be reported. Firstly, we want to stress that all samples come from only one center, Charité—Universitätsmedizin Berlin. Secondly, the study was retrospectively performed and lacks information regarding tumor recurrence and disease-free survival. Third, no DNA-sequencing data were available in the study cohort. It is of potential clinical interest to see if there are any associations between the type of *KRAS* or *TP53* mutations and the expression of the various immune checkpoints. Fourthly, post-translational modifications (e.g., glycosylation) may influence the binding between ICs and the corresponding antibodies used in this study and can potentially lead to false-negative results [24]. Such potential false-negative results can be detected using multiple antibodies for each marker and by removing post-translational modifications before staining. This will be addressed in future studies of a less explorative nature.

These results open multiple interesting avenues of exploration for future studies. We believe that there is a subtle interplay between immune cells, tumor cells, and nerve cells in PDAC. To maximize the potential of anticancer immunotherapy in the future, factors complementary to ICs that influence the complex interplay of immune cells in the TME need to also be taken into account [25,26]. We hypothesize that the VISTA pos./PD-L1 pos. subgroup is characterized also by specific subtypes of mutations in *TP53* and *KRAS*, and by a specific TME defined by various types of specialized immune cells and regulatory nerve fibers [27]. We will address these points in subsequent studies.

## 4. Materials and Methods

### 4.1. Patient Cohort

In this retrospective, single-center study approved by the institutional ethics committee (#EA2/031/21), samples from formalin-fixed, paraffin-embedded (FFPE) specimens of 68 patients with histologically confirmed PDAC—who underwent oncologic pancreatic resection between September 2009 and September 2020 in the Department of Surgery, Campus Benjamin Franklin, Charité—Universitätsmedizin Berlin—were included following informed consent. Of 93 patients, five patients that died within 30 days postoperatively, nine patients with metastatic disease at the time of surgery, seven patients with neuroendocrine tumors (NET) in the final histopathology report, two patients with tumors of duodenal origin, and two patients that had no follow-up after surgery as they were not living in Germany were excluded from this study (*n* = 25). Patients’ demographic and clinicopathological characteristics are presented in Table 1. The follow-up of patients in terms of OS was performed until death or for a median of 61 months (range: 36–118 months, censored cases, referred to as “survivors” in Results).

### 4.2. Surgery

All patients underwent oncologic pancreatic resection (pylorus-preserving pancreaticoduodenectomy, Whipple procedure, left-sided pancreatic resection or total pancreatectomy) according to the current guidelines after surgery was indicated by an interdisciplinary tumor board. One patient received neoadjuvant chemotherapy prior to tumor resection.

### 4.3. Tissue-Microarrays and Immunohistochemistry

FFPE specimens of resected PDAC were collected from the archive of the Institute of Pathology at the Charité—Universitätsmedizin Berlin, Campus Mitte and Campus Benjamin Franklin, Berlin, Germany. For the purpose of this study, tumor specimens were reviewed by two study pathologists (S.S. and M.P.D.) with 6 and 2 years of experience, respectively, in the histopathology of the pancreas, regarding cancer subtype, grading, pTNM-classification, vascular, lymphatic, and perineural invasion according to the 8th edition of the TNM classification (AJCC). They annotated the tumor area and excluded non-tumor regions, including necrosis and artefacts. For tissue microarray (TMA) construction, from each tumor, a total of six 2 mm tissue cores were punched out from high-tumor-purity regions of the tumor, three of them from different areas of the periphery of the tumor, and three from different areas of the tumor center; these were embedded in empty recipient paraffin blocks. For IHC analysis, the TMA blocks were cut into 4 μm sections. The sections were incubated in a CC1 mild buffer (Ventana Medical Systems, Tucson, AZ, USA) for 30 min at 100 °C or in protease 1 for 8 min. Afterwards, the sections were stained with an anti-B7H4 antibody (D1M8I, Cell Signaling, Danvers, Massachusetts, United States, 1:300), anti-IDO antibody (D5J4E, Cell Signaling, 1:100), anti-LAG3 antibody (D2G40; 1:300; Cell Signaling Technology), anti-PD-L1 antibody (E1L3N, Cell Signaling, 1:200), anti-TIM-3 antibody (D5D5R; 1:100; Cell Signaling Technology), and anti-VISTA antibody (D1L2G; 1:100; Cell Signaling Technology) for 60 min at room temperature, before being visualized using the avidin–biotin complex method and DAB. For this purpose, the BenchMark XT immunostainer (Ventana Medical Systems, Tucson, AZ, USA) was used. A detailed list of these applied antibodies is presented in Supplementary Table S1. For the counterstaining of cell nuclei, sections were incubated with hematoxylin and bluing reagent (Ventana Medical Systems, Tucson, AZ) for 12 min. In this way, for each patient, six IHC stainings from the respective TMA were prepared for IHC evaluation (three from the center and three from the periphery of each tumor). As usual in TMA processing, there is a certain loss of stainings (~10%). In our cohort, the overall median of stainings for each IC per patient was 6.0 (range 0–6, total of 2321 stainings). One patient had no IHC staining of VISTA, and three patients had no IHC staining of B7H4. The stainings were analyzed using an Olympus BX50 microscope (Olympus Europe). Histological images were acquired with the digital slide scanner, PANNORAMIC 1000 (3DHISTECH).

### 4.4. Evaluation of Immunohistochemistry and Threshold Selection

Evaluations of all IHC stainings were performed individually by the two study pathologists. The tumor cells on each staining were evaluated in terms of presence of the ICs: TIM-3, IDO, B7H4, IDO, LAG3, VISTA and PD-L1. For each respective IC, the ratio of visible positive cancer cells to total cancer cells per staining in percentage (%) was calculated. The tumor of a patient was classified as IC-positive if the respective IC was visible in at least 1% of cancer cells on at least one staining of the respective case. Discrepancies between the two study pathologists were discussed, and if no consensus was found, a third pathologist was consulted. Subsequently, to analyze the increasing expression levels, four subgroups were formed: ICs were visible in ≥ 1%, ≥ 1% and <10%, ≥ 10% and <50% and ≥ 50% of total cancers cells. The tumor of a patient was assigned to one of these subgroups when an IC was visible in the respective percentage of total cancers per staining on at least one of staining of the respective case (Supplementary Table S2). For ICs that significantly correlated with OS, intratumoral heterogeneity was evaluated via an analysis of distribution of expressed ICs across the different spots of the TMA of each individual tumor, generally, and regarding tumor center vs. tumor periphery.

### 4.5. Statistics

For our statistical analysis, we utilized SPSS version 28.0 (IBM Corp. Released 2021. IBM SPSS Statistics for Windows, Version 28.0. Armonk, NY, USA: IBM Corp.) Overall-survival (OS) Kaplan–Meier curves were plotted and compared using the log-rank test. For medians of OS, 95% confidence intervals (CI) were calculated. Cox proportional-hazards regression model was used for the calculation of univariate and multivariate hazard ratios for OS. Univariate variables with a trend towards significance (*p* < 0.10) and after testing of the proportional hazards assumption were used for analysis in the multivariate model. For the analysis of intratumoral heterogeneity of IC expression, we averaged the respective IC expression of all spots located in the periphery and in the center of the respective tumor and performed a paired t-test to test for differences. A *p*-value of <0.05 was considered a statistically significant difference. Graphics were designed using CorelDRAW^®^ Graphic Suite 2021 (Corel Corporation, Ottawa, ON, Canada) and GraphPad Prism Version 9.3.1 (GraphPad Software, San Diego, CA, USA).

## 5. Conclusions

The results of our study suggest that the presence of VISTA and PD-L1 in cancer cells of PDAC is of prognostic relevance and potentially qualifies them as targets for ICT. Further studies investigating the exact interplay between immune cells, tumor cells and nerve cells in PDAC, as well as their mutational subtypes in the TME, are warranted.

## Figures and Tables

**Table 1:** Demographic and clinicopathological characteristics.

Patients	*n* = 68
**Age**	
Median age (years)	72
Age range	35–86
**Sex**	
Female	30 (44.1%)
Male	38 (55.9%)
**Location of main tumor mass**	
Pancreatic head	55 (80.9%)
Pancreatic body	1 (1.5%)
Pancreatic tail	8 (11.8%)
Overlapping	4 (5.9%)
**Histopathological characteristics**	
pT1	3 (4.4%)
pT2	16 (23.5%)
pT3	49 (72.1%)
pN0	13 (19.1%)
pN+ (pN1 andand pN2)	55 (80.9%)
R0	56 (82.4%)
R1	12 (17.6%)
G1	2 (2.9%)
G2	39 (57.4%)
G3	26 (38.2%)
G4	1 (1.5%)
PN0	19 (27.9%)
PN1	49 (72.1%)
pL0	34 (50.0%)
pL1	34 (50.0%)
pV0	47 (69.1%)
pV1	21 (30.9%)

R: resection margin; G: grading; PN: perineural invasion; pL: lymphatic vessel invasion; pV: angioinvasion.

**Table 2:** Univariate and multivariate hazard ratios of demographic and pathological characteristics for OS.

	Univariate			Multivariate		
	HR	CI 95%	*p*-Value	HR	CI 95%	*p*-Value
Age (>65 vs. <65 years)	1.95	0.98–3.87	0.06	1.96	0.96–4.01	0.06
Sex (male vs. female)	1.49	0.86–2.57	0.15			
T stage (pT3–4 vs. pT1/2)	1.57	0.83.2.99	0.17			
Nodal involvement (pN+ vs. pN0)	5.09	2.01–12.92	<0.01	4.06	1.56–10.6	<0.01
Resection margin (R1 vs. R0)	3.00	1.50–5.60	<0.01	2.16	1.06–4.43	0.04
Grading (G3–4 vs. G1–2)	1.37	0.80–2.34	0.25			
Perineural invasion (Pn1 vs. Pn0)	1.61	0.88–2.96	0.13			
Lymphatic vessel invasion (pL1 vs. pL0)	1.87	1.09–3.20	0.02	1.21	0.66–2.21	0.55
Angioinvasion (pV1 vs. pV0)	2.52	1.43–4.43	<0.01	2.30	1.24–4.28	<0.01

HR: hazard ratio; CI 95%: 95% confidence interval.

**Table 3:** Presence of the ICs in all patients, the subgroup of survivors and subgroup of non-survivors.

IC	All Patients	Survivors	Non-Survivors
TIM3	16.2% (*n* = 11)	7.7% (*n* = 1)	18.2% (*n* = 10)
IDO	41.2% (*n* = 28)	30.8% (*n* = 4)	43.6% (*n* = 24)
B7H4	22.1% (*n* = 15)	15.4% (*n* = 2)	23.6% (*n* = 13)
LAG3	20.6% (*n* = 14)	15.4% (*n* = 2)	21.8% (*n* = 12)
VISTA	26.5% (*n* = 18)	7.7% (*n* = 1)	30.9% (*n* = 17)
PD-L1	33.8% (*n* = 23)	15.4% (*n* = 2)	38.2% (*n* = 21)
Any IC	66.2% (*n* = 45)	61.5% (*n* = 8)	67.3% (*n* = 37)

**Table 4:** Presence of the ICs of the formed subgroups and their respective one-year, three-year and five-year survival rates.

ICs	All Patients	One-Year SR	Three-Year SR	Five-Year SR
VISTA neg., PD-L1 neg.	52.9% (*n* = 36)	77.8%	38.9%	26.9%
VISTA pos., PD-L1 neg.	11.8% (*n* = 8)	25.0%	25.0%	12.5%
VISTA neg., PD-L1 pos.	19.1% (*n* = 13)	46.2%	15.4%	15.4%
VISTA pos., PD-L1 pos.	14.7% (*n* = 10)	50.0%	0.0%	0.0%

SR: survival rate.

**Table 5:** Expression of VISTA in the periphery, the center or both regions of tumors and respective survival rates.

Localization of VISTA Expression	Patients	Log-Rank *p*-Value	Median Survival No Expression vs. Expression
Periphery only	22.2% (*n* = 4)	*p* = 0.97	19 months (CI: 11.5–26.5) vs. 11 months (CI: 0.0–24.7)
Center only	44.4% (*n* = 8)	*p* = 0.01	19 months (CI: 12.8–25.2) vs. 4 months (CI: 0.0–9.5)
Periphery and center	22.2% (*n* = 4)	*p* < 0.01	19 months (CI: 13.4–24.6) vs. 2 months

## Data Availability

The datasets used and/or analyzed during the current study are available upon reasonable request pending approval by the local data security authorities.

## Supplementary Materials

The following supporting information can be downloaded at: https://www.mdpi.com/article/10.3390/ijms24032160/s1.
