# Supplementary material for: The Immune Checkpoint Landscape in Tumor Cells of Pancreatic Ductal Adenocarcinoma
Source: Int J Mol Sci. 2023 Jan 21;24(3):2160. doi: 10.3390/ijms24032160 (PMC9917344; doi:10.3390/ijms24032160)
Supplement: Supplementary file 1 [file ijms-24-02160-s001.zip › ijms-2079389-supplementary.pdf]

Supplementary Table S1. Applied antibodies for immunohistochemical staining.

Antigen	Abbreviation	Clone	Manufacturer	Catalog #	Dilution
T-cell immunoglobulin and mucin-domain containing-3	TIM-3	D5D5R	Cell Signaling	45208	1:500
Indoleamine 2,3-dioxygenase	IDO	D5J4E	Cell Signaling	86630	1:100
V-set domain-containing T-cell activation inhibitor 1	B7H4	D1M8I	Cell Signaling	14572	1:300
Lymphocyte-activation gene 3	LAG3	D2G40	Cell Signaling	15372	1:100
V-domain Ig suppressor of T cell activation	VISTA	D1L2G	Cell Signaling	64953	1:200
Programmed death-ligand 1	PD-L1	E1L3N	Cell Signaling	13684S	1:200

Supplementary Table S2. Subgroup analysis for subgroups formed by incremental increase in cancer cells expressing the respective IC. For markers that showed significance in the survival analysis of any subgroup ($p<0.05$), median survival for no expression vs. expression was calculated for each subgroup for comparison.

IC	Patients with expression	Log-rank p-value	Median survival no expression vs. expression
**Presence of respective immune checkpoint (IC) in ≥ 1% of tumor cells**			
Any IC	n=45	p=0.315	
TIM3	n=11	p=0.619	
IDO	n=28	p=0.295	21 months (16.4-25.5) vs.<br>13 months (7.0-19.0)
B7H4	n=15	p=0.598	
LAG3	n=14	p=0.705	
**VISTA**	**n=18**	**p=0.007**	22 months (CI: 12.9-31.1) vs.<br>7 months (CI: 0.1-13.9)
**PDL1**	**n=23**	**p=0.038**	22 months (CI 10.2-33.8) vs.<br>11 months (CI: 5.1-16.9)

**Presence of respective immune checkpoint (IC) in ≥ 1% and <10% of tumor cells**			
Any IC	n=42	p=0.187	
TIM3	n=8	p=0.973	
IDO	n=25	p=0.370	22 months (CI: 14.7-29.7) vs.<br>13 months (CI: 9.9-24.1)
B7H4	n=10	p=0.872	
LAG3	n=10	p=0.190	
**VISTA**	**n=13**	**p<0.001**	20 months (CI: 10.4-29.6) vs.<br>7 months (0.0-16.4)
PDL1	n=17	p=0.241	19 months (CI: 11.0-27.0) vs.<br>13 months (CI: 4.9-21.1)
**Presence of respective immune checkpoint (IC) ≥ 10% and <50% of tumor cells**			
Any IC	n=24	p=0.741	
TIM3	n=5	p=0.543	
**IDO**	**n=8**	**p=0.016**	19 months (CI: 12.4-25.6) vs.<br>4 months (CI: 0.0-13.2)
B7H4	n=7	p=0.972	
LAG3	n=4	p=0.364	
VISTA	n=1	p=0.550	19 months (CI: 11.4-26.6) vs.<br>11 months (CI: 0.0-23.8)
PDL1	N=12	p=0.116	19 months (CI: 10.4-27.6) vs.<br>11 months (4.2-17.8)
**Presence of respective immune checkpoint (IC) in ≥ 50% of tumor cells**			
Any IC	n=7	p=0.191	
TIM3	n=0	-	
IDO	n=3	p=0.780	17 months (CI: 10.2-23.8) vs.<br>28 months (0.0-63.2)
B7H4	n=0	-	
LAG3	n=0	-	
VISTA	n=1	p=0.163	18 months (CI: 11.0-25.0) vs.<br>6 months (CI: n=1)
PDL1	n=3	p=0.231	19 months (CI: 11.1-26.9) vs.<br>11 months (6.2-15.8)
